# Effects of Exogenous Abscisic Acid on the Physiological and Biochemical Responses of *Camellia oleifera* Seedlings under Drought Stress

**DOI:** 10.3390/plants13020225

**Published:** 2024-01-13

**Authors:** Dayu Yang, Yongzhong Chen, Rui Wang, Yimin He, Xiaofan Ma, Jiancai Shen, Zhilong He, Hanggui Lai

**Affiliations:** 1 School of Tropical Agriculture and Forestry, Hainan University, Haikou 570228, China; alexyangdayu@gmail.com (D.Y.); 18037275660@163.com (Y.H.); mxf17637320477@163.com (X.M.); ssw727892476@163.com (J.S.); 2 Research Institute of Oil Tea Camellia, Hunan Academy of Forestry, Changsha 410004, China; chenyongzhong@hnlky.cn (Y.C.); wangrui102@163.com (R.W.); 3 National Engineering Research Center for Oil-Tea Camellia, State Key Laboratory of Utilization of Woody Oil Resource, Hunan Academy of Forestry, Changsha 410116, China

**Keywords:** exogenous ABA, drought stress, *Camellia oleifera*, oxidative stress, foliar spray

## Abstract

This study comprehensively investigates the physiological and molecular regulatory mechanisms of *Camellia oleifera* seedlings under drought stress with a soil moisture content of about 30%, where exogenous abscisic acid (ABA) was applied via foliar spraying at concentrations of 50 µg/L, 100 µg/L, and 200 µg/L. The results demonstrated that appropriate concentrations of ABA treatment can regulate the physiological state of the seedlings through multiple pathways, including photosynthesis, oxidative stress response, and osmotic balance, thereby aiding in the restructuring of their drought response strategy. ABA treatment effectively activated the antioxidant system by reducing stomatal conductance and moderately inhibiting the photosynthetic rate, thus alleviating oxidative damage caused by drought stress. Additionally, ABA treatment promoted the synthesis of osmotic regulators such as proline, maintaining cellular turgor stability and enhancing the plant’s drought adaptability. The real-time quantitative PCR results of related genes indicated that ABA treatment enhanced the plant’s response to the ABA signaling pathway and improved disease resistance by regulating the expression of related genes, while also enhancing membrane lipid stability. A comprehensive evaluation using a membership function approach suggested that 50 µg/L ABA treatment may be the most-effective in mitigating drought effects in practical applications, followed by 100 µg/L ABA. The application of 50 µg/L ABA for 7 h induced significant changes in various biochemical parameters, compared to a foliar water spray. Notably, superoxide dismutase activity increased by 17.94%, peroxidase activity by 30.27%, glutathione content by 12.41%, and proline levels by 25.76%. The content of soluble sugars and soluble proteins rose by 14.79% and 87.95%, respectively. Additionally, there was a significant decrease of 31.15% in the malondialdehyde levels.

## 1. Introduction

*Camellia oleifera* Abel., a woody oil crop belonging to the Theaceae family and native to China, is renowned for the high-quality oil extracted from its seeds [1]. The oil from *C. oleifera* fruits, rich in unsaturated fatty acids, particularly oleic and linoleic acids, stands out for its nutritional value [2]. The bioactive compounds in tea oil, such as vitamin E and polyphenols, impart significant antioxidant properties, making it widely used in industries like food, medicine, and cosmetics [3]. Additionally, the cultivation of *C. oleifera* plays an important role in maintaining biodiversity and preventing soil erosion [4,5]. Hence, its cultivation is vital not only for agricultural production, but also for ecological protection and sustainable development.

It adapts well to various soil types, thriving in acidic to neutral soils, and exhibits a certain tolerance to soil fertility and moisture conditions. Despite this, drought stress remains one of the major environmental pressures affecting its growth and yield. Under drought conditions, the chlorophyll content in the leaves of *Camellia* plants decreases, stomatal conductance is reduced, and photosynthesis efficiency is limited, leading to inhibited growth [6]. Drought stress also directly affects the development of *Camellia* fruits, subsequently impacting the yield and quality of tea oil [7].

In response to drought stress, plants activate their antioxidant systems, accumulate osmotic regulators, and adjust the accumulation of plant hormones to adapt to the arid environment. Drought stress can lead to an overproduction of reactive oxygen species (ROS) in plants. Plants mitigate this oxidative damage by activating antioxidant enzyme systems such as superoxide dismutase (SOD), catalase (CAT), and peroxidase (POD) [8,9]. Moreover, plants accumulate osmoregulatory substances like proline and soluble sugars, lowering cellular osmotic potential and retaining moisture. These substances not only help plants absorb and conserve water but also protect cell membranes and reduce oxidative stress [10].

In plant responses to abiotic stress, phytohormones play a crucial role, coordinating with reactive oxygen species to adapt to various abiotic stresses [11]. Exogenous application of hormones such as ABA [12], melatonin [13], jasmonic acid [14], epibrassinolide [15], and gibberellins [16] has been proven to effectively enhance plant adaptability to abiotic stresses. Among these, ABA is one of the primary hormones mediating the drought stress response. One key physiological reaction of plants to drought is the ABA-mediated stomatal closure, which maximizes the reduction of water loss due to transpiration before the relief of water stress [17]. Additionally, the significant increase in ABA levels under drought stress leads to the formation of ABA receptor complexes, subsequently inhibiting protein phosphatases (PP2Cs) and activating SNF1-related protein kinases (SNRK2s). The released SNRK2s can trigger downstream stress-related signal transduction processes [18].

Studies by Awan et al. [19] have shown that exogenous ABA treatment enhances the drought resistance of pearl millet. Experimental results indicated that plants treated with ABA maintained better growth and water status in simulated drought conditions, and reduced oxidative damage. Further research by Zhou et al. [20] suggested that ABA treatment could regulate the expression of specific proteins involved in glycolysis, stability of photosystem II, and other key biological processes against drought stress. However, in studies related to the response of *C. oleifera* plants to drought stress, literature on the application and mechanisms of exogenous ABA is relatively scarce, with most research focusing only on the physiological and biochemical responses of *C. oleifera* seedlings under drought stress. Therefore, this study aims to explore the effects of exogenous ABA applied via foliar spraying on *C. oleifera* seedlings under drought conditions. We will focus on the impacts of different concentrations of ABA treatment on the photosynthesis of seedlings, the antioxidant enzyme system, the accumulation of osmotic regulators, and the expression of related genes. This will not only enhance our understanding of the drought stress response in *C. oleifera* but also provide references for drought resistance management strategies for the plants.

## 2. Materials and Methods

### 2.1. Experimental Materials and Design

**Figure 1:**
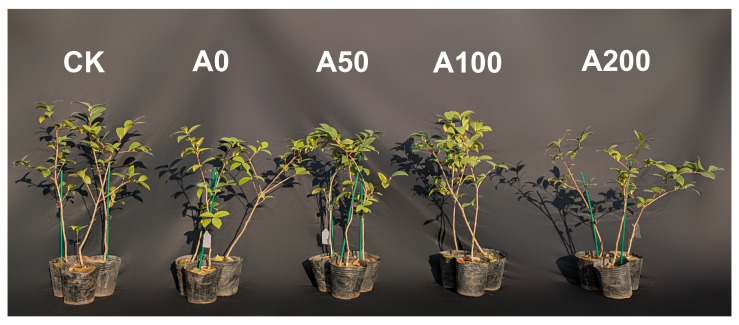
Experimental Layout of *C. oleifera* Seedlings Subjected to Different ABA Concentrations. The abbreviations and corresponding full names are as follows: CK: A normal growth group; A0: Group sprayed with water under drought stress; A50: Group sprayed with with 50 µg/L ABA under drought stress; A100: Group sprayed with with 100 µg/L ABA under drought stress; A200: Group sprayed with with 200 µg/L ABA under drought stress.

The experiment was conducted in 2023 at the Tianjiling Experimental Forestry Farm of the Hunan Academy of Forestry, China (113°01′ E, 28°06′ N), using healthy and uniformly grown two-year-old potted *C. oleifera* seedlings, specifically the Xianglin No.1 variety. Based on previous studies, the photosynthetic parameters of Xianglin No.1 deteriorated rapidly between 8 to 12 days of water withholding, suggesting that the 8th day after stopping watering (with soil moisture content around 30%) is a critical juncture for the recovery potential of *C. oleifera* seedlings [21]. Therefore, in this study, after sufficient watering, the seedlings were subjected to water withholding to simulate drought conditions. On the 8th day (with soil moisture content around 30%), the seedlings were treated with exogenous ABA through foliar spraying in the morning at 7 a.m. In determining the concentrations of ABA for our experiment, we referred to prior studies which demonstrated that a concentration of 100 µg/L ABA significantly affects the net photosynthetic rate, transpiration rate, and *LHY* gene expression in Camellia seedlings under a photoperiod of medium day length [22]. Consequently, we selected 50 µg/L, 100 µg/L, and 200 µg/L as our experimental concentrations.The experiment consisted of five groups, as shown in Figure 1: a normal growth group (CK), group sprayed with water (A0), and group sprayed with 50 µg/L (A50), 100 µg/L (A100), and 200 µg/L ABA (A200), with three *Camellia* seedlings in each group. To prevent cross-contamination among treatments, a distance of three meters was maintained between groups of seedlings during the foliar application of varying ABA concentrations. The photosynthetic parameters of the seedlings were measured at 8 a.m. (T1), 11 a.m. (T2), and 2 p.m. (T3), and leaves were randomly collected at each time point and immediately stored in liquid nitrogen for subsequent physiological and biochemical analysis.

### 2.2. Measurement of Photosynthetic Parameters

The net photosynthetic rate (Pn), intercellular CO_2_ concentration (Ci), stomatal conductance (Gs), and transpiration rate (Tr) of *Camellia* seedlings functional leaves were randomly measured using a 6400XT Portable Photosynthesis System (LI-COR, Lincoln, NE, USA). The airflow rate of the system was set to 400 μ mol/s, and the intensity of photosynthetically active radiation (PAR) was set to $800\;\mu {\mathrm{mol}/m}^{2}/s$ .

### 2.3. Determination of Physiological and Biochemical Indices

In this study, the activities of POD and SOD, as well as the contents of malondialdehyde (MDA), glutathione (GSH), proline (Pro), soluble sugars (SS), and soluble proteins (SP), were determined using kits purchased from Ruixin Bio-Tech Co., Ltd. (Ruixinbio, Quanzhou, China).

### 2.4. Membership Function Method

In this study, a membership function method was employed to comprehensively evaluate multiple indices, assessing the alleviation effect of different exogenous ABA concentrations [23]. Initially, the membership function values for each index under different ABA concentration treatments were calculated. Subsequently, the sum of the membership function values of all indices in each group was averaged (D) to assess the effectiveness of different ABA concentration treatments. The effectiveness of each ABA concentration treatment was determined based on the magnitude of the D value. The formulas for calculating the membership function values are as follows:

(1) $$u\left(X_{ij}\right)=\frac{X_{ij}-X_{jmin}}{X_{jmax}-X_{jmin}}$$

(2) $$u\left(X_{ij}\right)=1-\frac{X_{ij}-X_{jmin}}{X_{jmax}-X_{jmin}}$$

In these formulas, $u(X_{ij})$ represents the membership function value of index *j* under treatment *i*, $X_{ij}$ is the measured average value of index *j* under treatment *i*, $X_{jmax}$ is the maximum value of index *j* across all treatments and $X_{jmin}$ is the minimum value of index *j* across all treatments. Formula (1) is used when an index positively correlates with drought resistance, while Formula (2) is used when an index negatively correlates with drought resistance. The calculation of membership function values was carried out in Excel.

### 2.5. RNA Extraction and Real-Time Fluorescent Quantitative Analysis

Total RNA from *Camellia* seedlings leaves was extracted using the RNAprep Pure Plant Plus Kit (TIANGEN, Beijing, China), and the first strand cDNA was synthesized using the HiScript III 1st Strand cDNA Synthesis Kit (+gDNA wiper) (Vazyme, Nanjing, China). The cDNA was then diluted five times to serve as a template for real-time fluorescent quantitative PCR (qRT-pcr), with tubulin as the reference gene. The qRT-pcr reagents used were ChamQ Universal SYBR qPCR Master Mix (Vazyme, Nanjing, China). Primers required for the qRT-pcr experiment were designed using Primer Premier 5 software (Premier Biosoft, Palo Alto, CA, USA), and their sequences are shown in Table 1. The $2^{-\Delta \Delta C_{t}}$ method was used to calculate the relative transcription levels of the genes.

### 2.6. Statistical Analysis

In this study, data collection and preliminary processing were conducted using Microsoft Excel 2021 software (Microsoft Corporation, Redmond, WA, USA). The statistical analysis of the experimental data was performed using IBM SPSS Statistics 27 software (IBM Corporation, Armonk, NY, USA). To assess the significant differences in photosynthetic parameters and physiological indicators among various treatment groups, an analysis of variance (ANOVA) was initially conducted, followed by Duncan’s multiple range test to elucidate the disparities between groups. In the presentation of results, distinct uppercase letters indicate significant differences between treatment groups at the same time point, while lowercase letters denote significant variations within the same treatment group across different time points. The expression levels of key genes at each time point (T1, T2, T3) were analyzed using one-way ANOVA. For each time point, the differences between each treatment group and the control group (CK) were assessed using Tukey’s Honest Significant Difference (HSD) test. A *p*-value of less than 0.05 was considered statistically significant. Graphs and charts were generated using GraphPad Prism 9 software (GraphPad Software, San Diego, CA, USA).

## 3. Results

### 3.1. Effects of Exogenous ABA on Photosynthetic Parameters of *C. oleifera* Seedlings under Drought Stress

**Figure 2:**
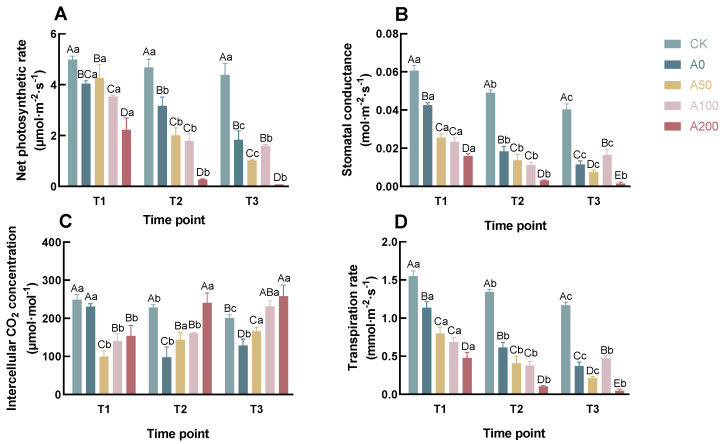
Effects of Exogenous ABA on the Pn (**A**), Gs (**B**), Ci (**C**), and Tr (**D**) of *C. oleifera* Seedlings Leaves under Drought Stress. According to Duncan’s multiple range test, Each bar is labeled with both uppercase and lowercase letters to indicate statistical significance. Uppercase letters denote significant differences between the means of different treatments at the same time point (for example, `A’ differs from `B’ signifies (*p* < 0.05)), while lowercase letters signify significant differences over time within the same treatment (for example, `a’ at T1 versus `b’ at T2 signifies (*p*< 0.05)). Vertical bars represent the standard deviation of the mean (*n*= 3). The abbreviations and corresponding full names are as follows: T1: 8 a.m.; T2: 11 a.m.; T3: 2 p.m.

As illustrated in Figure 2A, the control group (CK) maintained relatively stable net photosynthetic rates (Pn), with only minor fluctuations over time. In stark contrast, the drought-treated groups experienced a pronounced decline in Pn. Specifically, at T3, the Pn of the A50, A100, and A200 groups decreased by 44.28%, 12.53%, and 95.50%, respectively, compared to the A0 group. While the A50 group exhibited a marginal increase in Pn by 5.38% at T1 compared to A0, this difference was not statistically significant ( p>0.05 ). This suggests that exogenous ABA application under drought stress can significantly impacts Pn in seedlings, with the extent of photosynthetic inhibition varying with ABA concentrations. Remarkably, the Pn value in the A200 group dropped drastically to a very low level at T2, potentially dueto the excessive suppression of photosynthesis at high ABA concentrations. Interestingly, the Pn decrease in the A100 group was notably slower at T3.

Figure 2B,D depict that the patterns of Gs and Tr are strikingly similar across all groups, both demonstrating a consistent decline over time. Significantly lower values of Gs and Tr were observed in the drought-treated groups compared to the control (CK) group ( p<0.05 ). At T3, the Gs values in the A50 and A200 groups dropped by 33.92% and 87.43%, respectively, relative to the A0 group, while the A100 group experienced a 43.86% increase in Gs. Correspondingly, Tr values in the A50 and A200 groups fell by 42.35% and 87.73%, respectively, compared to A0, with the A100 group showing a 27.87% increase. Notably, at T2, the A200 group demonstrated a significant decline in both Gs and Tr, paralleling the reduction in Pn. The notable rise in Gs and Tr values in the A100 group at T3 compared to A0 ( p<0.05 ) may account for the marked slowdown in Pn reduction at this juncture.

The variation in Ci, as depicted in Figure 2C, indicates a more intricate scenario. In the CK and A0 groups, a general decreasing trend in Ci values was observed over time. In stark contrast, plants subjected to ABA treatment treatment demonstrated a progressive increase in Ci. At T1, the Ci level in the A0 group was significantly higher than that in the A50, A100, and A200 groups ( p<0.05 ). However, as time progressed, the Ci in the A0 group sharply declined at T2, remaining lower than those in the ABA-treated groups at both T2 and T3. The CK group exhibited a more gradual decrease in Ci, reflecting stability. Among the ABA treatment groups, there was a consistent rise in Ci with increasing ABA concentrations at each time point. These changes in Ci may reflect the complex interplay between stomatal activity and plant adaptive responses to drought stress.

### 3.2. Effects of Exogenous ABA on Physiological Indicators of *C. oleifera* Seedlings under Drought Stress

**Figure 3:**
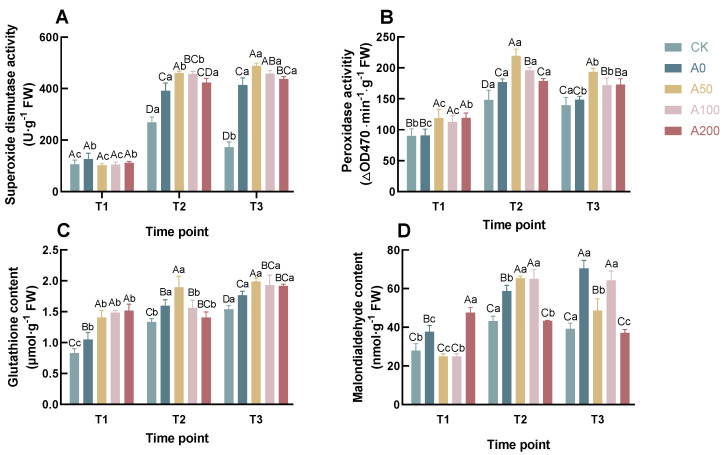
Effects of Exogenous ABA on the Antioxidant System of *C. oleifera* Under Drought Stress, Including SOD (**A**), POD (**B**), GSH (**C**), and MDA (**D**). According to Duncan’s multiple range test, Each bar is labeled with both uppercase and lowercase letters to indicate statistical significance. Uppercase letters denote significant differences between the means of different treatments at the same time point (for example, `A’ differs from `B’ signifies (*p* < 0.05)), while lowercase letters signify significant differences over time within the same treatment (for example, `a’ at T1 versus `b’ at T2 signifies (*p*< 0.05)). Vertical bars represent the standard deviation of the mean (*n*= 3). The abbreviations and corresponding full names are as follows: T1: 8 a.m.; T2: 11 a.m.; T3: 2 p.m.

As the duration of treatment increased, the SOD activity in the CK groupinitially rose and then fell, whereas in the drought-treated groups, it consistently remained elevated at T3 (Figure 3A). Notably, at both T2 and T3 the SOD activity in the ABA-treated groups surpassed that in the A0 group. Specifically, at T3, the SOD activity in the A50, A100, and A200 groups increased by 17.94%, 10.52%, and 5.60%, respectively, compared to A0. The overall trend of POD activity initially exhibited an increase followed by a decrease, as depicted in Figure 3B. Concurrently, an escalating ABA concentration corresponded to a gradual reduction in POD activity, particularly evident at T2 and T3. The A0 group’s POD activity at T1 and T3 did not significantly differ from CK, except at T2, where it recorded a 19.33% increase over CK. In contrast, the A50 and A100 groups consistently displayed significantly higher POD activity than A0 across all time points ( p<0.05 ), while the A200 group exhibited notably higher activity than A0 at T1 and T3 ( p<0.05 ), but not at T2 compared to CK2 ( p>0.05 ). At T3, the increases in POD activities for A50, A100, and A200 were 30.27%, 15.70%, and 16.37%, respectively, in relation to A0. These findings indicate that exogenous ABA application under drought stress significantly enhances the activities of SOD and POD, pointing to its vital role in modulating antioxidative responses in *C. oleifera* seedlings.

The dynamics of MDA content, an indicator of lipid peroxidation, exhibited complexity in its pattern. In the CK group, the MDA content showed an initial increase followed by a decrease, maintaining lower concentrations throughout all three time points (Figure 3D). Conversely, in the A0 group, MDA content showed a steady increase over the duration of treatment. Within the ABA-treated groups, MDA levels in A50 and A100 did not differ significantly at T1 and T2 ( p>0.05 ). At T3, the MDA content in A50 and A100 decreased by 31.15% and 8.85%, respectively, compared to A0. The A200 group exhibited a downward trend in overall MDA levels, presenting significantly higher content at T1 than A0 ( p<0.05 ), but notably lower at T2 and T3 ( p<0.05 ). These These observations imply that exogenous ABA application can effectively mitigate lipid peroxidation in *Camellia* seedlings, as evidenced by the reduction in MDA content.

The GSH content across all groups displayed an general upward trend (Figure 3C), with levels in the drought-treated groups surpassing those in the CK group. At T1, no significant difference was observed among the ABA-treated groups, yet all exhibited substantially higher GSH content than both A0 and CK ( p<0.05 ). At T1, the GSH content in A50, A100, and A200 groups increased by 33.77%, 41.44%, and 44.26%, respectively, compared to A0. By T3, these increases were 12.41%, 9.29%, and 8.51% respectively. This pattern suggests that exogenous ABA application under drought stress conditions significantly boosts the GSH content in the leaves of *Camellia* seedlings.

**Figure 4:**
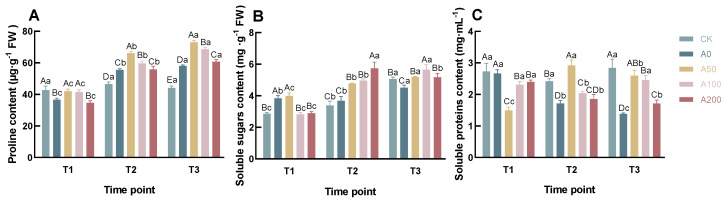
Effects of Exogenous ABA on the Content of Pro (**A**), SS (**B**), and SP (**C**) in *C. oleifera* Seedlings Under Drought Stress. According to Duncan’s multiple range test, Each bar is labeled with both uppercase and lowercase letters to indicate statistical significance. Uppercase letters denote significant differences between the means of different treatments at the same time point (for example, `A’ differs from `B’ signifies (*p* < 0.05)), while lowercase letters signify significant differences over time within the same treatment (for example, `a’ at T1 versus `b’ at T2 signifies (*p*< 0.05)). Vertical bars represent the standard deviation of the mean (*n*= 3). The abbreviations and corresponding full names are as follows: T1: 8 a.m.; T2: 11 a.m.; T3: 2 p.m.

The Pro content in the CK group remained stable and relatively low, showing no significant variation ( p>0.05 ) across the three time points (Figure 4A). In contrast, a gradual increase in Pro levels was observed in all drought-treated groups. Notably, at each time point, the Pro content in both A50 and A100 was significantly higher than that in A0 ( p<0.05 ), while the A200 exhibited no significant difference from A0 at T1 and T2, but a marked increase at T3 ( p<0.05 ). At T3, the Pro content in A50, A100, and A200 increased by 25.76%, 18.24%, and 4.61%, respectively, compared to A0. This indicates that exogenous ABA application under drought stress conditions significantly elevates the Pro content in the leaves of *Camellia* seedlings, with a notable observation that Pro accumulation tends to decrease with increasing ABA concentration.

The content of SS exhibited an overall increasing trend across all groups (Figure 4B). At T1, the SS levels in the CK and A50 groups were similar, and significantly higher than those in other groups ( p<0.05 ). At the T2 and T3, the SS contents in A50, A100, and A200 groups were significantlyelevated compared to both A0 and CK ( p<0.05 ). Notably, at T3, the increases in SS content for A50, A100, and A200 were 14.79%, 25.28%, and 14.63%, respectively, in relation to A0.These observations suggest that treatment with exogenous ABA under drought stress modifies the accumulation pattern of SS in *Camellia* seedlings’ leaves, enabling a rapid upsurge in response to drought.

The pattern of SP varied considerably among the groups (Figure 4C). In the CK group, SP content remained stable and at higher levels throughout the three time points. In both A0 and A200 groups, SP levels initially declined, then stabilized at lower levels by T3, while the A50 group showed an initial increase followed by stabilization at a steady level. The A100 group presented a trend of initial decrease followed by an increase, with SP content at T3 being comparable to that of A50. At T3, the SP content in A50, A100, and A200 increased by 87.95%, 77.71%, and 23.88%, respectively, compared to A0.This indicates that the application of exogenous ABA under drought stress significantly influences SP content in *Camellia* seedlings’ leaves, with varied responses among different treatment groups.

### 3.3. Effects of Exogenous ABA on Gene Expression in *C. oleifera* Seedlings under Drought Stress

**Figure 5:**
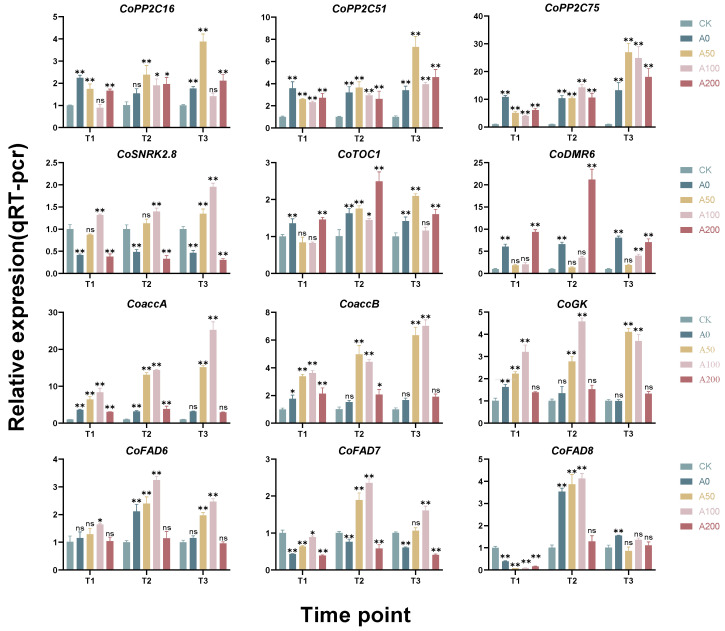
Effects of Exogenous ABA on the Expression of Key Genes in *C. oleifera* Under Drought Stress, Including *CoPP2C16*, *CoPP2C51*, *CoPP2C75*, *CoSNRK2.8*, *CoTOC1*, *CoDMR6*, *CoaccA*, *CoaccB*, *CoGK*, *CoFAD6*, *CoFAD7*, and *CoFAD8*. Statistical significance at each time point, determined by one-way ANOVA followed by Tukey test, is indicated relative to the CK on the bars. Asterisks denote significant differences compared to CK: * *p* < 0.05, ** *p* < 0.01. `ns’ indicates no significant difference.Vertical bars represent the standard deviation of the mean (*n* = 3). The abbreviations and corresponding full names are as follows: *CoPP2C16*: Protein Phosphatase 2C 16; *CoPP2C51*: Protein Phosphatase 2C 51; *CoPP2C75*: Protein Phosphatase 2C 75; *CoSNRK2.8*: Sucrose Non-Fermenting 1 (SNF1)-Related Protein Kinase 2.8; *CoTOC1*: Timing of CAB expression 1; *CoDMR6*: Downy mildew resistant 6; *CoaccA*: Acetyl-coenzyme A carboxylase carboxyl transferase subunit alpha; *CoaccB*: Biotin carboxyl carrier protein; *CoGK*: Glycerol kinase; *CoFAD6*: Fatty Acid Desaturase 6; *CoFAD7*: Fatty Acid Desaturase 7; *CoFAD8*: Fatty Acid Desaturase 8; T1: 8 a.m.; T2: 11 a.m.; T3: 2 p.m.

In this study, to gain a deeper understanding of the impact of exogenous ABA on the physiological and biochemical aspects of *Camellia* seedlings under drought stress, CoTubulin was used as the reference gene. The expression levels of key genes in the leaves of *Camellia* seedlings, including *CoPP2C16*, *CoPP2C51*, *CoPP2C75*, *CoSNRK2.8*, *CoTOC1*, *CoDMR6*, *CoaccA*, *CoaccB*, *CoGK*, *CoFAD6*, *CoFAD7*, and *CoFAD8*, were analyzed through quantitative PCR (qPCR) (Figure 5).

The results showed that the three genes of the PP2C family, *CoPP2C16*, *CoPP2C51*, and *CoPP2C75*, were significantly upregulated in the A0 group compared to the CK group. At the T1 time point, the expression of these three genes was suppressed to varying degrees in the A0, A100, and A200 groups compared to A0. In the A50 group at T3, the expression levels of *CoPP2C16*, *CoPP2C51*, and *CoPP2C75* were upregulated by 119.81%, 114.46%, and 103.54%, respectively, compared to A0. In the A100 group, the expression levels of *CoPP2C16* and *CoPP2C51* showed little difference from A0 at T2 and T3, while *CoPP2C75* was upregulated by 87.85% at T3. The expression levels of *CoPP2C16*, *CoPP2C51*, and *CoPP2C75* in the A100 group at T3 were upregulated by 20.01%, 34.50%, and 36.34%, respectively, compared to A0.

The *CoSNRK2.8* gene in the A0 group was suppressed compared to CK at all three time points. In the ABA-treated groups, *CoSNRK2.8* in the A50 and A100 groups showed a significant upregulation compared to A0 at all three time points, with a trend of gradual increase. At the T3 time point, the expression levels of the *CoSNRK2.8* gene in the A50 and A100 groups increased by 188.26% and 317.15%, respectively, compared to A0. There was little difference between A200 and A0 across all three time points

The *CoTOC1* gene in the A0 group was significantly upregulated compared to CK at all three time points. In the A50 group, *CoTOC1* expression was downregulated at T1 compared to A0, then significantly upregulated at T3. In the A100 group, *CoTOC1* expression was lower than A0 at all three time points, while in the A200 group, it showed no significant difference from A0 at T1 and T3, and was significantly higher than A0 at T2.

The *CoDMR6* gene in the A0 group exhibited significant upregulation compared to CK across all three time points. Interestingly, in the ABA-treated groups, the *CoDMR6* gene displayed a pattern of suppression at low concentrations and enhancement at high concentrations. Notably, the expression of the *CoDMR6* gene in both the A0 and A50 groups was significantly suppressed relative to A0 during all three time points. Furthermore, in the A200 group, the expression of the *CoDMR6* gene was markedly upregulated compared to A0 at T1 and T2. This trend was especially pronounced at T2, where the expression of *CoDMR6* increased by a striking 221.93% compared to A0.

Genes related to lipid synthesis, such as *CoaccA*, *CoaccB*, *CoGK*, *CoFAD6*, *CoFAD7*, and *CoFAD8* tended to be upregulated in the A50 and A100 groups compared to A0, while in the A200 group, the expression of these genes showed little change compared to A0. Notably, the expression of *CoaccA*, *CoaccB*, and *CoGK* in the A50 and A100 groups was significantly upregulated at all three time points, showing a trend of gradual increase. The genes encoding fatty acid desaturases, *CoFAD6*, *CoFAD7*, and *CoFAD8*, showed an initial increase followed by a decrease, peaking at T2, with a significant upregulation compared to A0.

### 3.4. Comprehensive Evaluation through Membership Function Analysis

To determine the optimal concentration of exogenous ABA for alleviating drought stress in *C. oleifera*, this study employed a comprehensive evaluation method using membership function analysis. By assessing multiple parameters such as Pn, Gs, Tr, Ci, SOD, POD, MDA, GSH, Pro, SP, and SS, the study calculated the overall scores (D values) for each treatment to identify the most effective concentration of exogenous ABA.

As can be inferred from Table 2, the D values ranked from highest to lowest are A50, A100, A200, and A0, respectively. This may suggest that in practical applications, treating *Camellia* seedlings with 50 µg/L ABA could potentially yield the best mitigation effects against drought, followed by the 100 µg/L ABA treatment.

## 4. Discussion

### 4.1. Photosynthetic Parameters

Under drought stress conditions, the photosynthetic mechanisms of plants are significantly impacted, primarily manifested in damage to the photosynthetic electron transport chain, reduced activity of ribulose-1,5-bisphosphate carboxylase/oxygenase, and decreased chlorophyll content. These changes directly lead to a significant reduction in the net photosynthetic rate [24,25,26]. In our study, we observed that exogenous ABA treatment further inhibited the photosynthetic rate, contradicting the findings of Hu et al. [27], but aligning with the conclusions of Chen et al. [28] and Vahideh Khaleghnezhad et al. [29]. The varying effects of exogenous ABA on Pn might depend on factors such as plant species, the severity of drought stress, concentration, and frequency of ABA application. On one hand, plants often exhibit a decrease in Pn as a response to oxidative attacks on cellular lipid membranes by ROS and the direct impact of water scarcity, potentially as a physiological strategy to adapt to water stress. On the other hand, the further reduction in Pn due to exogenous ABA treatment may reflect ABA’s role in regulating plant energy metabolism, where the plant sacrifices some photosynthetic efficiency to reconfigure energy metabolic pathways for more effective drought stress adaptation.

Significantly, our study observed that the A50 group maintained a relatively higher Pn, potentially due to an optimal balance between stomatal regulation and CO_2_ absorption at this specific ABA concentration. In contrast, the A200 group exhibited a marked decrease in Gs and Tr, implying that higher ABA concentrations may lead to excessive stomatal closure, thus substantially inhibiting Pn. Intriguingly, the decline in Pn for the A100 group at T3 was less evident compared to T2, a trend that might be associated with the observed increase in Gs and Tr from T2 to T3.

Our study also found that exogenous ABA treatment significantly reduced GS but observed an increase in Ci, particularly at higher ABA concentrations. This phenomenon indicates that the reduction in Pn under ABA treatment might result from a combination of stomatal and non-stomatal factors. Non-stomatal limitations could include reduced activity of photosynthetic enzymes and decreased efficiency of the photosynthetic electron transport chain, leading to inefficient photosynthesis even in the presence of adequate CO_2_ [30,31]. Therefore, our research suggests that while ABA treatment reduces stomatal conductance to minimize water loss, it might also lower the photosynthetic efficiency of *C. oleifera* by altering internal regulatory mechanisms of photosynthesis. This dual effect allows *Camellia* plants to prioritize survival over growth under drought stress, thereby enhancing its adaptability to drought conditions.

### 4.2. Physiological Indicators

Under abiotic stress conditions, SOD acts as the first line of defense against ROS damage, converting them into O_2_ and H_2_O_2_. Subsequently, CAT and POD can further metabolize H_2_O_2_, thereby mitigating oxidative stress in plants [32,33]. Our study results are consistent with previous findings, showing that at suitable concentrations, ABA treatment significantly enhances the activities of SOD and POD in the leaves of *Camellia* seedlings [34]. Reduced GSH in the cells can eliminate ROS through non-enzymatic reactions, serving as a key antioxidant substance [35]. In our study, we found that ABA treatment significantly increased the GSH content in *Camellia* seedlings. This might be due to the enhancement of GSH synthesis or the glutathione cycle in plants after ABA treatment [36,37]. MDA, a product of cellular membrane lipid peroxidation, can react with proteins and damage DNA structures and is widely regarded as an important indicator of the extent of lipid peroxidation damage [38,39]. Our data shows that ABA treatment significantly reduces the MDA content in *Camellia* seedlings, indicating that the antioxidant system in the ABA treatment group effectively reduces ROS, thereby minimizing membrane lipid damage.

Osmoregulatory substances such as proline and soluble sugars in plant cells help maintain osmotic balance and stabilize cell turgor under drought stress [40,41,42]. In our study, compared to CK and A0, the use of exogenous ABA under drought stress conditions significantly increased the content of proline and soluble sugars in leaves. Furthermore, we observed that the A200 group had the highest soluble sugar content at T2. This could be due to the plant adjusting its energy and metabolite allocation in response to reduced photosynthetic rates, increasing soluble sugar content by reducing sugar consumption and breaking down starch. On the other hand, previous research indicates that plants under stress conditions can induce the production of new proteins such as heat shock proteins, dehydration protective proteins, and signaling proteins, while some damaged proteins are tagged with ubiquitin and degraded through the ubiquitination pathway [43,44]. Therefore, the protein content in plants under stress is the cumulative result of the degradation of damaged proteins and the synthesis of new proteins. In our study, the soluble protein content in the A50 and A100 groups showed an overall upward trend and was significantly higher than A0 at T2 and T3, indicating that suitable concentrations of ABA can increase the content of soluble proteins in plants. This increase might be related to ABA’s role in regulating the transport of various proteins, carbon metabolism, and the expression of resistance proteins [20].

### 4.3. Gene Expression

In plants’ response to environmental stresses, the ABA signaling pathway plays a central role. Key components of this pathway include ABA receptors (PYR/PYL/RCAR), PP2Cs, and SNRK2s. Under drought stress, ABA molecules bind to their receptors (PYR/PYL/RCAR), leading to the inhibition of PP2Cs, which in turn activates SNRK2s. The activated SNRK2s initiate downstream ABA responses through phosphorylation, regulating drought resistance-related genes [45]. Previous studies [21] have shown that in two varieties *C. oleifera*, genes like *PP2C16*, *PP2C51*, *PP2C75*, and *SNRK2.8* play a crucial regulatory role through the ABA signaling pathway under drought stress. It has been reported that the overexpression of the *SNRK2.8* gene in poplar significantly enhances its resistance to salt and drought stress [46]. In our study, we found that after the application of exogenous ABA, the expression levels of *PP2C16*, *PP2C51*, and *PP2C75* were downregulated at T1 compared to A0, which may help amplify the signal, allowing SNRK2s to be rapidly activated in the early stages. Subsequently, at T3, their expression levels increased compared to A0, possibly reflecting a negative feedback adjustment of the signal’s strength and sensitivity. This change might facilitate the activation of more SNRKs to regulate downstream drought resistance-related genes. The continuous increase in the expression of *SNRK2.8* in the A50 and A100 groups further confirms this. This suggests that under drought stress conditions, suitable concentrations of exogenous ABA can effectively enhance ABA signal transduction.

In this work, we found that ABA treatment significantly altered the expression patterns of genes such as *CoTOC1* and *CoDMR6*. In Arabidopsis, *TOC1* has been reported to be involved in the ABA response pathway under drought stress through the ABA receptor (ABAR) [47]. Research by Henrique suggests that the expression of *TOC1* in the aerial parts of plants is crucial for maintaining adaptability to drought conditions. Tobacco plants with *TOC1* deficiency showed reduced leaf water loss and improved water use efficiency under drought conditions, though their total seed capsule production significantly decreased [48]. In our study, the expression of *CoTOC1* in the ABA treatment groups was significantly affected, implying that exogenous ABA might enhance the drought resistance of *Camellia* seedlings by regulating *CoTOC1*. We also observed significant variations in the expression of *CoDMR6* under different ABA concentration treatments. The silencing of the *DMR6* gene has been demonstrated to significantly enhance disease resistance in various plants, including tomatoes [49], potatoes [50], and grapevine [51]. In our study, compared to A0, the expression of *CoDMR6* was inhibited in the low-concentration ABA treatment groups (A50, A100), while it showed an upregulation trend in the high-concentration ABA treatment group (A200). This variation in expression patterns might reflect the complex physiological strategies plants adopt to cope with drought stress, where appropriate ABA concentrations not only improve drought adaptability but may also enhance disease resistance by suppressing the expression of *CoDMR6*. However, at higher ABA concentrations, due to reduced photosynthetic efficiency, plants may reduce the allocation of resources to non-essential stress responses. Additionally, the differential changes in *CoDMR6* expression in the ABA treatment groups could also indicate the gene’s sensitivity to ABA concentrations.

It has been reported that under drought stress, a higher degree of fatty acid unsaturation is crucial for maintaining the stability of plant membrane fluidity [52]. Overexpression of fatty acid desaturases *FAD3* and *FAD8* has been demonstrated to significantly improve the tolerance of tobacco plants to abiotic stress [53]. Additionally, mounting evidence suggests that the remodeling of plant lipids is one of the effective adaptive strategies against abiotic stress [54]. In our study, genes related to lipid synthesis, such as *CoaccA*, *CoaccB*, *CoGK*, *CoFAD6*, *CoFAD7*, and *CoFAD8*, were significantly upregulated in the A50 and A100 groups, indicating the important role of ABA treatment in reshaping plant membrane lipids. Indeed, ABA treatment has been shown to induce changes in plant lipids, thereby improving drought adaptability in various plants including wheat [55], tea plants [56], maize [52], and mosses [57]. This might be because ABA treatment can regulate genes associated with lipid synthesis, enhance the degree of fatty acid unsaturation, and effectively alleviate the attack of ROS on polyunsaturated fatty acids in membrane lipids.

### 4.4. Limitations and Future Prospects

The current study preliminarily demonstrates that exogenous ABA can enhance the drought adaptability of potted *Camellia* seedlings. However, due to the limitations of pot experiment conditions, these findings need to be validated through more extensive and long-term field trials. Future research should consider various factors such as different *C. oleifera* cultivars, growth stages of seedlings, and severity of drought conditions to determine the optimal concentration for ABA application. Additionally, the role of other substances like exogenous melatonin and exogenous calcium in enhancing plant drought resistance suggests the potential for combined treatments in the drought resistance management of *Camellia* plants. Despite these limitations, the application of exogenous ABA has already shown positive effects in improving the drought adaptability of *Camellia* seedlings.

## 5. Conclusions

This study comprehensively investigated the effects of foliar-applied exogenous ABA on the physiological and molecular regulatory mechanisms of *C. oleifera* seedlings under drought stress. It was found that appropriate concentrations of ABA treatment could regulate the physiological state of the seedlings through multiple pathways, including photosynthesis, oxidative stress response, and osmotic balance. This not only helps to ensure plant survival but also maintains basic growth activities to a certain extent. The application of exogenous ABA effectively reduced stomatal conductance and moderately inhibited the photosynthetic rate, while significantly activating the antioxidant system, thus alleviating oxidative damage caused by drought stress. Furthermore, ABA treatment promoted the synthesis of osmoregulatory substances like proline, effectively maintaining cellular turgor stability and enhancing the plant’s adaptability to adverse conditions. Analysis of gene expression revealed that ABA treatment increased the plant’s sensitivity to the ABA signaling pathway, improved membrane lipid stability, and potentially enhanced disease resistance by regulating the expression of related genes. A comprehensive evaluation using the membership function method suggests that under drought stress conditions in practical production of *Camellia* plants, the application of 50 µg/L ABA is possibly the most effective, followed by 100 µg/L ABA.

## Figures and Tables

**Table 1:** Gene primer sequences used for the quantitative real-time PCR analysis.

Gene	Forward Primer (5′-3′)	Reverse Primer (5′-3′)
*CoTubulin*	CCATGCCTTGGATCACATTT	TGGGGCCATTAATGTAGACG
*CoPP2C16*	GGAGGCAAGGTGATTCAGTGG	GGCATTCGTCTTCTTTCGTTCG
*CoPP2C51*	CAGGGTGTTTGGTGTTCTTTCC	CTCATCCTCGTCACTCCTTGTC
*CoPP2C75*	TTCTCGGTGTTCTCGCTATGTC	GTCGCTCGCCAGTATCAGG
*CoSNRK2.8*	GCATTCCCAACCCAAATCTACAG	GCACCAACCAACATCACATATAAGG
*CoTOC1*	AGGAGAAGGCGAATGCTTGG	CTCTACAGGAGCAGCAGCAG
*CoDMR6*	TCAAGGATGGCAAGTGGATGG	GGCAGAGGAAAGAGGCTATGG
*CoaccA*	CGAAACCGGTCTGGACTTCA	CTCGATCTCCGTGAAGCTCC
*CoaccB*	CCACACCACCACCTATTCCC	TCCAGAAGCCTCCAATGCTG
*CoGK*	AGTTGAGTCCACTGGCGGAGTT	ACAGCACGAGCGATGTGAGACT
*CoFAD6*	GGCTCAGCTCAATGGCACAGTT	CGCCAATTCCAAGTCGCCTCAT
*CoFAD7*	ATCATGGCATCCGTTGTCTGA	GGTCTTCCCAGGACTTCTACCC
*CoFAD8*	TCAATGGCGTCAATGGGG	CGGAATCGCAGCTCGAATAT

The abbreviations and corresponding full names are as follows: *CoTubulin*: Tubulin alpha-3 chain; *CoPP2C16*: Protein Phosphatase 2C 16; *CoPP2C51*: Protein Phosphatase 2C 51; *CoPP2C75*: Protein Phosphatase 2C 75; *CoSNRK2.8*: Sucrose Non-Fermenting 1 (SNF1)-Related Protein Kinase 2.8; *CoTOC1*: Timing of CAB expression 1; *CoDMR6*: Downy mildew resistant 6; *CoaccA*: Acetyl-coenzyme A carboxylase carboxyl transferase subunit alpha; *CoaccB*: Biotin carboxyl carrier protein; *CoGK*: Glycerol kinase; *CoFAD6*: Fatty Acid Desaturase 6; *CoFAD7*: Fatty Acid Desaturase 7; *CoFAD8*: Fatty Acid Desaturase 8.

**Table 2:** Comprehensive Evaluation Table Using Membership Function Analysis.

Group	Membership Function Value											D	Rank
	**Pn**	**Gs**	**Tr**	**Ci**	**SOD**	**POD**	**MDA**	**Pro**	**GSH**	**SP**	**SS**		
A0	1	0.67	0.24	0	0	0	0	0	0	0	0	0.17	4
A50	0.54	0.41	0.61	0.29	1	1	0.66	1	1	1	0.59	0.73	1
A100	0.87	1	0	0.8	0.59	0.52	0.19	0.71	0.75	0.88	1	0.66	2
A200	0	0	1	1	0.31	0.54	1	0.18	0.68	0.27	0.58	0.51	3

The values for each parameter in the table represent the membership function values, and the D value is the average of the membership function values for each group. The abbreviations and corresponding full names of relevant indicators are as follows: Pn: Net photosynthetic rate; Gs: Stomatal conductance; Tr: Transpiration rate; Ci: Intercellular CO_2_ concentration; SOD: Superoxide dismutase; POD: Peroxidase; MDA: Malondialdehyde; Pro: Proline; GSH: Glutathione; SP: Soluble proteins; SS: Soluble sugars.

## Data Availability

Data are available on request from the corresponding authors.
